# Supplementary material for: Chronic exposure to environmental temperature attenuates the thermal sensitivity of salmonids
Source: Nat Commun. 2023 Dec 14;14:8309. doi: 10.1038/s41467-023-43478-7 (PMC10721842; doi:10.1038/s41467-023-43478-7)
Supplement: Supplementary file 1 — Supplementary Information [file 41467_2023_43478_MOESM1_ESM.pdf]

SUPPLEMENTARY INFORMATION

# *Chronic exposure to environmental temperature attenuates the thermal sensitivity of salmonids*

Alexia M. González-Ferreras[1,2*], Jose Barquín[1], Penelope S.A. Blyth[3,4], Jack Hawksley[3], Hugh Kinsella[2,5], Rasmus Lauridsen[6,7], Olivia F. Morris[3], Francisco J. Peñas[1], Gareth E. Thomas[2,8], Guy Woodward[3], Lei Zhao[9], & Eoin J. O'Gorman[2]

[1] *IHCantabria - Instituto de Hidráulica Ambiental de la Universidad de Cantabria. C/Isabel Torres 15, 39011 Santander, Spain*

[2] *School of Life Sciences, University of Essex, Wivenhoe Park, Colchester, CO4 3SQ, UK*

[3] *Georgina Mace Centre for the Living Planet, Department of Life Sciences, Imperial College London, Silwood Park Campus, Buckhurst Road, Ascot, SL5 7PY, UK*

[4] *School of Biosciences, University of Sheffield, Sheffield, S10 2TN, UK*

[5] *Trinity College Dublin, Ireland*

[6] *Game & Wildlife Conservation Trust, Salmon and Trout Research Centre, BH20 6BB, UK*

[7] *Six Rivers Iceland, Reykjavik, 101, Iceland*

[8] *Department of Life Sciences, Natural History Museum, Cromwell Road, London SW7 5BD, UK*

[9] *Beijing Key Laboratory of Biodiversity and Organic Farming, College of Resources and Environmental Sciences, China Agricultural University, Beijing, 100193 China*

***Corresponding author:** gferrerasam@unican.es

**Table S1: Number of fish individuals captured by electrofishing including in the field respirometry per experimental context and per specie.** Mean weight (in g) and standard deviation are also indicated.

| Specie | Experimental context | $N_{initial}$ | Mean Weight (g) | SD |
|---|---|---|---|---|
| Brown trout | SW Iceland_$_{AC}$ | 83 | 19.827 | 13.037 |
| Brown rout | SW Iceland_$_{CH}$ | 90 | 51.416 | 51.777 |
| Brown trout | UK_$_{CH}$ | 188 | 8.23 | 3.208 |
| Brown trout | Spain_$_{CH}$ | 93 | 22.47 | 26.244 |
| Salmon | Spain_$_{CH}$ | 22 | 9.995 | 9.715 |
| Salmon | NE Iceland_$_{CH}$ | 35 | 11.486 | 4.678 |

**Table S2: Parameter estimates in our previous work[1] for the bioenergetics model used in the Hengill streams.**

| Parameter | Lower 95% CI | Estimate | Upper 95% CI |
|---|---|---|---|
| $A_{G2}$ | 3.158 | 4.097 | 4.385 |
| $A_{G3}$ | 7.079 | 7.079 | 7.079 |
| $A_{H2}$ | -7.241 | -7.241 | -7.241 |
| $A_{H3}$ | 2.332 | 8.995 | 10.354 |
| $C_{G2}$ | -1.056 | -0.453 | -0.327 |
| $C_{G3}$ | 1.002 | 1.002 | 1.002 |
| $C_{H2}$ | -1.002 | -1.002 | -1.002 |
| $C_{H3}$ | -2.985 | -0.210 | 0.538 |
| $A_K$ | 17.491 | 18.082 | 18.657 |
| $E_K$ | -0.468 | -0.304 | 0.200 |

**Table S3: Statistical output of multiple linear regression model** describing the relationship between routine metabolic rate [ln($I$) in mg O2 h$^{-1}$] as the response variable and fish body mass [ln($M$) in mg], standardised Arrhenius temperature ($T_A$ in K), and the source stream that the fish were collected from ($S$; with 3 levels: IS1, IS12 and IS5) as explanatory variables.

| **Effects** | **DF** | **Sum of squares** | **Mean square** | ***F* value** | ***p* value** |
|---|---|---|---|---|---|
| ln($M$) | 1 | 17.060 | 17.060 | 61.999 | <0.001 |
| $T_A$ | 1 | 5.631 | 5.631 | 20.463 | <0.001 |
| $S$ | 2 | 0.689 | 0.344 | 1.252 | 0.292 |
| Residuals | 74 | 20.362 | 0.275 | | |

**Figure S1: Length-weight relationship used to obtained individual weights (g) of brown trout (Hengill system- SW Iceland_$_{AC}$) and Atlantic salmon (NE Iceland_$_{CH}$).** a) Linear regression $y = 3.02x - 5.00$, $F_{1,457} = 2.76{\times}10^4$, $p < 0.001$, $r^2 = 0.98$, and b) Linear regression $y = 2.69x - 4.40$, $F_{1,62} = 1448$, $p < 0.001$, $r^2 = 0.96$. Note that the relationships were constructed from empirical length and weight data collected at the very same sites.

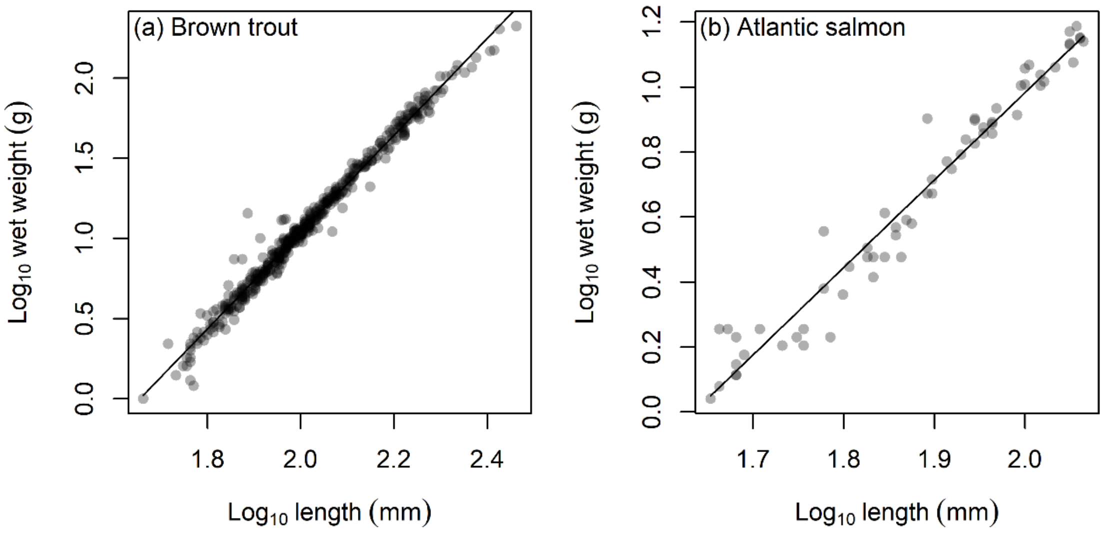

**Figure S2: Photographs taken during field sampling for illustrative purposes.** a) Control chamber, b) fish respiration chamber, c) top view of chambers and d) frontal view of chambers placed into the water with rock on the top. See methods in the main text for more information.

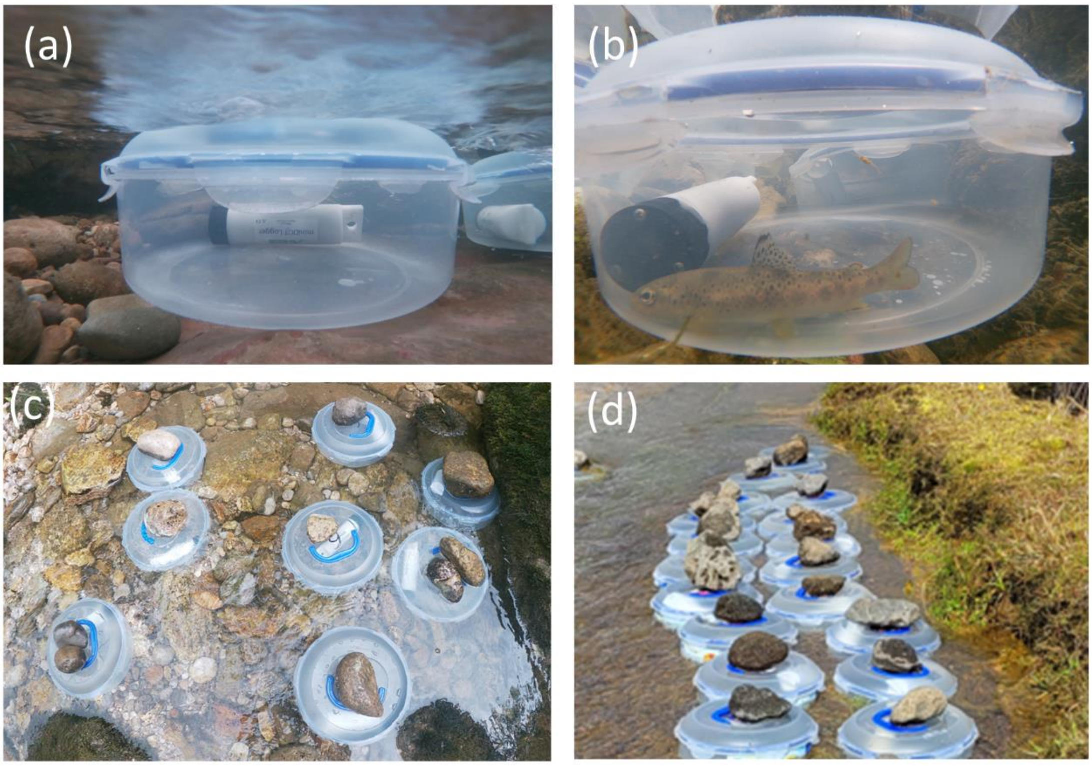


## References

1. O’Gorman, E. J. *et al.* Unexpected changes in community size structure in a natural warming experiment. *Nat Clim Chang* **7**, 659–663 (2017).
